# Light pollution in complex ecological systems

**DOI:** 10.1098/rstb.2022.0351

**Published:** 2023-12-18

**Authors:** Myriam R. Hirt, Darren M. Evans, Colleen R. Miller, Remo Ryser

**Affiliations:** ^1^ German Centre for Integrative Biodiversity Research (iDiv) Halle-Jena-Leipzig, Puschstr. 4, 04103 Leipzig, Germany; ^2^ Institute of Biodiversity, Friedrich-Schiller-University Jena, Jena, 07743, Germany; ^3^ School of Natural and Environmental Sciences, Newcastle University, Newcastle upon Tyne, NE1 4LB, UK; ^4^ Department of Ecology & Evolutionary Biology, Cornell University, Ithaca, NY, 14853, USA; ^5^ Cornell Laboratory of Ornithology, Ithaca, NY, 14850, USA

**Keywords:** artificial light at night, community, ecosystem, modelling, network, global change

## Abstract

Light pollution has emerged as a burgeoning area of scientific interest, receiving increasing attention in recent years. The resulting body of literature has revealed a diverse array of species-specific and context-dependent responses to artificial light at night (ALAN). Because predicting and generalizing community-level effects is difficult, our current comprehension of the ecological impacts of light pollution on complex ecological systems remains notably limited. It is critical to better understand ALAN's effects at higher levels of ecological organization in order to comprehend and mitigate the repercussions of ALAN on ecosystem functioning and stability amidst ongoing global change. This theme issue seeks to explore the effects of light pollution on complex ecological systems, by bridging various realms and scaling up from individual processes and functions to communities and networks. Through this integrated approach, this collection aims to shed light on the intricate interplay between light pollution, ecological dynamics and humans in a world increasingly impacted by anthropogenic lighting.

This article is part of the theme issue ‘Light pollution in complex ecological systems’.

## 1. Introduction

Organisms of most forms have adapted to using light as a source of energy or information. Primary producers such as plants, algae and cyanobacteria use light to synthesize complex molecules, grow and store energy. The overwhelming majority of organisms also use light as a carrier of information. For instance, light allows many organisms to process information about the abiotic and biotic environment. Moreover, variability in the light regime works like a metronome for many organisms by determining the timing and duration of days, months (or moon cycles) and seasons. For example, some plants use this information to time the onset of developing leaves or flowers [1], while corals have been shown to align the time point of spawning with the moon [2–4] and nocturnal animals usually start their daily activity after solar irradiance drops below a certain level [5]. Furthermore, some animals use light for orientation. For example, sea turtle hatchlings find their way to the ocean by moving towards the brighter horizon, which under natural conditions is over the ocean [6] and some birds and other animals can even navigate by starlight [7,8]. Overall, this highlights that natural light cycles are of immense importance for life on Earth.

The natural light regime, however, was disrupted when humans began to artificially light the night by using fire from wood, candles and oil lamps. The pollution of the night sky was recognized for the first time by astronomers in the first half of the twentieth century [9] as it started to interfere with their celestial observations and this phenomenon soon attracted the attention of a wider audience [10]. While artificial light at night (ALAN) is crucial for the everyday life of humans, Bara & Falchi [11] point out that only a tiny fraction of reflected light is used for human vision, while the rest pollutes the environment. With an estimated annual average increase of 9.6% (estimated based on citizen science data), light pollution is one of the most pressing drivers of current global change [12], and it has become increasingly clear that the loss of the night has serious psychological, health, socio-economic and ecological consequences.

From a biological perspective, natural light regimes, in contrast to other environmental conditions such as temperature, have remained more or less consistent over aeons of Earth's history. This makes light pollution a novel perturbation to which organisms are unlikely to have evolved the ability to cope with change [13–15]. Thus, it is not surprising that ALAN has the potential to fundamentally disrupt physiology and behaviour of many organisms. Famously, Shakespeare originated the phrase ‘like a moth to a flame’ in his 1605 play, *The Merchant of Venice*, noting the innate desire of some insects to draw close to light. Recent work attributes this phenomenon to the insects' natural behaviour of angling their back towards the brightest visual hemisphere, which results in continuous steering around artificial light sources [16]. Such behavioural changes in turn have consequences from ecological functions and processes to communities and ecosystems, thereby threatening biodiversity and ecosystem functioning.

Compared to the impacts of light pollution on the physiology and behaviour of individual species, which show a variety of context-dependent responses [17], the effects within more complex ecological systems (e.g. communities and ecological networks) remain relatively understudied ([18], but see [19–22]). Given the inherent complexity of natural systems, this knowledge gap limits our ability to predict the consequences of light pollution for ecosystems and the invaluable services they offer. While there are many other understudied areas that deserve increased attention in light biology (such as evolutionary implications), adopting a complex systems approach while also considering the different properties of light, such as different spectral compositions or light intensities (i.e. the amount of light), is important to develop more targeted mitigation strategies to reduce the negative consequences of ALAN for both humans and nature.

Therefore, this issue aims to provide new insights into the effects of ALAN on complex ecological systems, offering a foundation for a mechanistic understanding that facilitates upscaling from processes and functions to higher levels of ecological organization, which will also foster our predictive capabilities. Additionally, it addresses the properties of light with an emphasis on the relatively unexplored realm of skyglow, and the impact of different spectral compositions. Lastly, these papers shed light on the complex interplay between ALAN and humans, including its potential impact on vector-disease dynamics. Overall, the issue seeks to further our understanding of the impacts of ALAN in complex ecological systems, while providing directions for future research.

## 2. Properties of light

**Figure 1.**
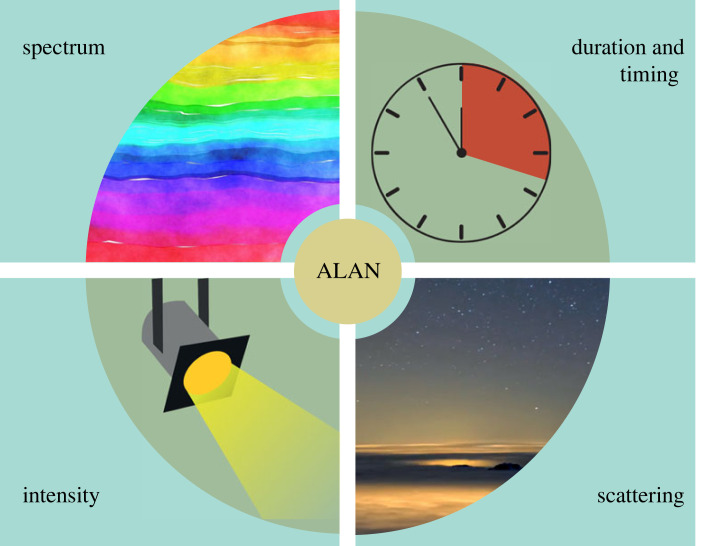
The properties of light. Artificial light possesses several physical characteristics that are crucial to understanding its impact on ecosystems, as well as to formulating informed mitigation strategies. These characteristics include: (i) spectral composition (which can have varying effects on species and ecosystems), (ii) duration and timing (e.g. the lengths of illumination periods during the night, or the precise timing of illumination), (iii) intensity (i.e. amount of light), and (iv) scattering. Light tends to become more scattered through processes such as reflection by aerosols, which typically decreases its intensity. This phenomenon is most prominent in the so-called ‘skyglow’. Images for ‘spectrum’, ‘intensity’ and ‘duration and timing’ ©pixabay. Image for ‘scattering’ ©Alessandro Della Bella. (Online version in colour.)

Compared to other global change drivers, such as temperature, artificial light has several properties that are relevant to understanding its impacts on ecological systems [23]. These include differences in spectral composition, intensity (i.e. amount of light), scattering and temporal aspects (e.g. duration or timing of illumination) (figure 1).

With the advent of electrification in the 1880s, light from fire was replaced with the invention of the incandescent electric and later gas discharge lamp. In the past two decades, the revolution in light-emitting diode (LED) technology is once again changing the way people light up the night, commonly replacing high-pressure sodium street lamps. All of these lighting technologies have different spectra, with current LED lighting often peaking in the shorter blue wavelengths that have been shown to trigger behavioural responses in many taxa as well as in people [24,25]. Thus, research into how different spectra affect ecological systems [26,27] is crucial for informed mitigation strategies, not least as LEDs can be easily engineered to different intensities and spectral compositions. In this issue, Parkinson & Tiegs [28] show that during short-term illumination in riparian systems, particular spectra of LED lighting can change the flux of resources between aquatic and terrestrial systems as well as the community composition of insects. Interestingly, Spoelstra *et al*. [29] demonstrate an effect of long-term illumination on terrestrial invertebrate community composition independent of light spectra. This could hint at spectral-specific response times to artificial light in the short term that level out in the long run.

Another important aspect of light to be considered, particularly in regard to potential mitigation strategies, is the duration and timing of illumination. This includes, for example, switching on light based on motion sensors or only illuminating part of the night [30]. Heinen *et al*. [31] look at the effects of short-term reduction of illumination time (i.e. turning lights off during parts of the night), which has been proposed as a potential mitigation strategy. However, they show an increased negative effect on aphid population growth when lights are temporarily switched off during the night compared to night-long illumination. Thus, ALAN can have both positive and negative effects at the population level, confirming the need for wider encompassing studies.

Since adaptations in lighting strategies (e.g. changes in spectral composition and reduction in illumination time) could be used to help mitigate the effects of ALAN on networks of interacting species, as pointed out by Evans [32], it is crucial to study the ecological effects of the properties of light.

Other ecologically relevant characteristics of ALAN are the intensity (e.g. radiance or luminance, irradiance or illuminance etc.) and scattering of light, which can be inter-related. While point-light sources such as street lamps are the most studied form of ALAN, a much more widely extended ALAN phenomenon is the so-called ‘skyglow’. Skyglow is the artificial light that is scattered (i.e. diverted) back to Earth by molecules, aerosols and clouds within the atmosphere [33]. Therefore, it is the spatially most widespread and thus likely an ecologically highly relevant form of light pollution, although it is usually much dimmer than nearby point sources. Since many organisms are adapted to using low-intensity light cues from star- and moonlight, low-intensity light pollution probably already has profound effects on biological processes. Some community-level research in this issue reveals considerable effects of skyglow. For instance, Bucher *et al*. [34] demonstrate reductions in plant biomass and diversity as well as changes in plant traits (e.g. leaf hairiness) in a grassland plant community exposed to low-intensity diffuse illuminance. In this experiment, Dyer *et al*. [35] report an increase in invertebrate movement activity at night, which is linked to higher predation rates. Cesarz *et al*. [36] also report significant effects on soil communities and functioning (in this case, soil basal respiration and carbon use efficiency). However, Grenis *et al*. [37] reveal contrasting effects on community composition of moths in response to skyglow and point-light sources. Moreover, Barrientos *et al*. [38] show that mountain lions are affected by direct light sources rather than skyglow, suggesting that avoiding potential interactions with humans is important for these animals in their selection of light landscapes. Overall, these findings underscore the necessity of differentiating between skyglow and point-light sources in future research endeavours.

## 3. Light pollution in complex ecological systems

Light pollution research has predominantly focused on studying the effects of artificial lighting on individual behaviour and physiology [17,39]. Numerous studies have shown impacts on, for example, movement patterns, activity, hormone levels and reproductive behaviour [17]. For instance, disruption of natural orientation [40,41] as well as changes in activity patterns [42,43] have been observed in many species. Moreover, by disrupting the natural circadian rhythms of animals, artificial light can lead to imbalances in hormone production and regulation [44,45] with severe consequences for growth, reproduction and overall fitness.

While significant progress has been made in elucidating physiological and behavioural responses to ALAN, our knowledge of community responses remains limited [18,46]. Because virtually all organisms are embedded in communities within ecosystems, therefore directly and indirectly influenced by species interactions, the responses of ecosystems are more than the sum of individual organism's responses. In consequence, an understanding of the effects of ALAN at higher organizational levels is essential for predicting how biodiversity and ecosystem services ultimately respond. Recognizing the importance of adopting a more community-centred and ecosystem-wide perspective, researchers have begun to shed light on this area (e.g. [21,47–50]). However, there is still much work to be done to systematically understand community-level responses to light pollution across ecosystem types. Research in this issue demonstrates significant effects of ALAN on community composition of grassland plant communities [34], terrestrial above- [29,37] and belowground [36] as well as freshwater invertebrate communities [51]. Going beyond such ecosystem boundaries, Parkinson & Tiegs [28] conducted their research at the intersection of aquatic and terrestrial systems. Their results propose that in such systems, ALAN could lead to asymmetrical resource fluxes, in terms of both insect abundance and biomass. This highlights the importance of species interactions interconnecting light effects across realms as well as different levels of ecological organization.

**Figure 2.**
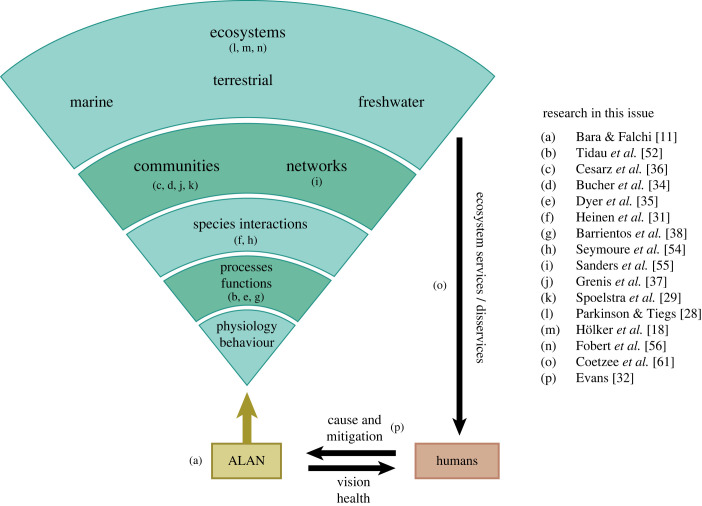
Effects of ALAN on complex ecological systems. ALAN propagates through different levels of ecological organization. Influencing individual physiology and behaviour, it alters key ecological processes and functions, such as movement or growth, resulting in significant changes to species interactions and the complex networks they form within and across communities. Consequently, the effects of ALAN cascade to entire ecosystems, leading to profound implications for ecosystem functioning. Humans as the cause of light pollution are inherently woven into the intricate web of these impacts. While they gain direct benefits from nocturnal illumination, they also experience negative consequences either directly through, e.g. health issues, or indirectly through a chain of ecosystem feedback effects that could potentially lead to a reduction in ecosystem services. (Online version in colour.)

Therefore, it is crucial to establish a connection between research on individual behaviour and physiology and the broader community-level effects (figure 2), as this linkage enables us to scale up our findings and gain deeper insights into the underlying mechanisms at play.

Notably, alterations in both physiology and behaviour can exert significant influence on fundamental ecological processes and functions [39]. Research in this issue reveals significant effects on growth and development of marine invertebrates [52], plant production [34], soil respiration [36] and movement activity and space use [35,38]. Such changes in processes and functions can have strong consequences for species interactions and ecological networks that constitute entire communities [53]. Specifically, Seymoure *et al*. [54] highlight the fast-growing realm of study in ALAN biology, by putting forth a framework to study species interactions under ALAN, while Heinen *et al*. [31] and Dyer *et al*. [35] both put this work into action in plant-insect community experiments. For example, Heinen *et al*. [31] report a largely indirect effect of ALAN on plant defence through herbivory, with ALAN altering herbivore population size which then upregulates plant defences. Dyer *et al.* [35] show that increased movement activity can increase predation rates probably via an increased encounter rate between predator and prey. Such changes in species interactions have far-reaching implications for ecological networks and their stability, determining which species can coexist and form communities within and across ecosystems (figure 2). In this issue, Sanders *et al*. [55] model pathways through which ALAN may alter species interactions, indicating that temporal niche shifts owing to the introduction of ALAN may rewire species networks and ecosystems, causing biodiversity change. Moreover, Bucher *et al*. [34] show a significant decrease in plant diversity. These findings, coupled with results demonstrating severe impacts on community composition, indicate profound implications for entire ecosystems and their functioning. For example, as emphasized in the review by Fobert *et al*. [56], substantial changes at the system level are anticipated in both temperate and tropical coral reefs. Moreover, Hölker *et al*. [51] discuss the possibility that responses to ALAN among or within taxonomic groups may create novel communities ‘with no historical analogues’. This would not only shift natural systems at an ecosystem level, but potentially have significant impacts on novel and adjacent communities near artificial light sources.

Overall, direct effects of ALAN on behaviour and physiology of various species can lead to a domino effect on entire communities and ecosystems. This, in turn, poses a threat to ecosystem functioning and services that humans depend on, as illustrated in figure 2. By connecting the different levels of ecological organization, we can advance our mechanistic understanding of the impacts of light pollution, which also enables predictions for consequences of ALAN in different contexts.

## 4. Modelling and predicting consequences of light pollution

**Figure 3.**
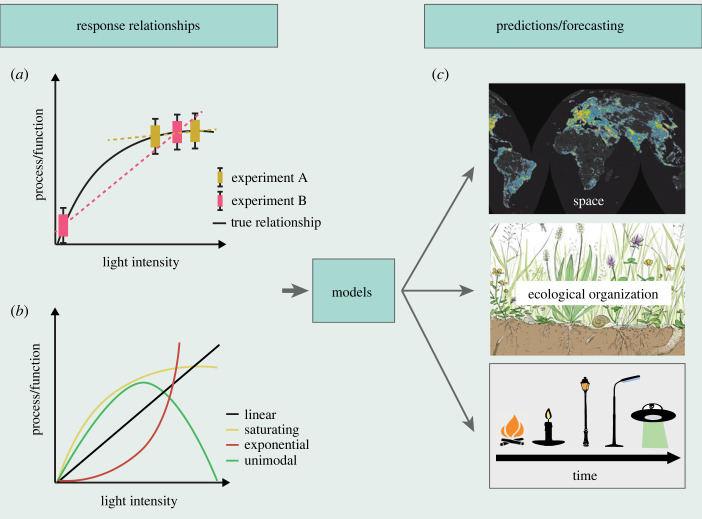
Modelling and predicting consequences of light pollution. (*a*) Studies that employ only two (e.g. presence/absence) or too few light levels (i) are at risk of misinterpreting the true impact of light and (ii) limit our ability to make predictions. In the example depicted here, light intensity is thought to have a saturating effect on a certain ecosystem process or function. However, experiment A (in yellow) would fail to capture any significant effect of ALAN, while experiment B (in pink) would overlook the saturating trend. Therefore, it is essential to select an adequate number of light levels to accurately capture the complex relationships between light and organisms (*b*). The insights gained from fully quantitative (e.g. dose–response) relationships provide the basis to formulating theories and developing models. These models can then be employed to better predict the consequences of ALAN across various dimensions: space, time and different levels of ecological organization (c). Image for ‘space’ from Falchi *et al*. 2016 [33]. Image for ‘ecological organization’ ©Phillip Janta. Images for ‘time’ ©pixabay. (Online version in colour.)

Gaining a mechanistic and general understanding of the impacts of ALAN on different levels of ecological organization, as described in the preceding section, provides the basis to formulating theories and developing models. These models can then be used to make better predictions of the consequences of ALAN across space and time. The term ‘prediction’ encompasses two key aspects: firstly, it involves predicting the effects of ALAN on species or areas that have not been directly measured, as well as under unexplored light conditions (figure 3). These predictions can also extend to forecasting the effects of ALAN under future light conditions. Secondly, it entails harnessing our understanding of how ALAN effects propagate through ecological systems (figure 2) to predict community- and ecosystem-level responses to ALAN.

Accurate predictions and extrapolations heavily depend on a thorough understanding of the mechanistic relationships between variables and the shape of those relationships. To achieve this level of understanding, it is essential to employ fully quantitative experiments (also referred to as ‘dose–response’) to gain insights into both the direction and the strength of the effects. For instance, a naturally continuous variable such as light intensity needs to be assessed across a representative range of intensities to understand the shape of the relationship and be able to make predictions [18]. Light intensity is one of the most important and most widely studied aspects of light pollution. However, the majority of studies on this topic focus on binary comparisons (the presence or absence of light) or include only a limited number of light intensity levels (but see [22,57]), probably owing to practical constraints in the feasibility of experiments, particularly at the community level [18]. Although these studies have provided a solid foundation for recognizing the significant impact of light pollution on organisms, it is important to bear in mind the limitations that accompany them.

One notable limitation pertains to the selection of light levels in the experiments, which can potentially obscure or overlook certain significant effects (as depicted in figure 3*a*). The chosen light levels might not fully capture the complexity of the relationship between light and organisms, especially when considering the possibility of nonlinear and intricate relationships. In this theme issue, for example, research by Tidau *et al*. [52] illustrates nonlinear relationships between irradiance and barnacle survival rate. Also, Dyer *et al*. [35] demonstrate a saturating relationship with a steep increase in invertebrate movement activity with light intensity, emphasizing that more than 50% of the change observed in their study already occurred at illuminance levels that were below that of a maximum full moon. The insights gained from such fully quantitative relationships provide the basis to formulating theories and developing models. Such models can then be employed to better predict the consequences of ALAN across space, time and different levels of ecological organization (figure 3c). In this issue, Sanders *et al*. [55] integrated a complex food web model with potential temporal niche shifts of diurnal, nocturnal and crepuscular species in response to ALAN. They show that the homogenization of temporal niches in particular can rewire species networks with varying consequences for species persistence.

Such predictions and forecasts of the effects of light pollution on complex ecological systems also hold significant importance as they provide a sandbox for testing and refining mitigation strategies.

## 5. Interconnection with humans: causes, consequences and mitigation

It is important to recognize that as humans, we are not only responsible for light pollution but also integrated within the network of its effects on ecosystems (figure 2). The introduction of artificial lighting, as highlighted by Bara & Falchi [11], has opened up the ‘nocturnal niche’ for human activities, offering benefits such as increased productivity, safety and convenience. However, alongside these advantages, artificial lighting also has direct negative effects on humans, including disruptions of the circadian rhythm, sleep disturbances and an increased risk of certain diseases like cancer [58].

Furthermore, the impacts of ALAN extend beyond direct effects on humans. Through its influence on ecological systems, ALAN can cause cascading indirect effects that have broader implications for human well-being. By altering ecological processes and functions in plants and animals, ALAN disrupts the functioning of entire ecosystems and the services they provide (e.g. pollination, pest control and nutrient cycling) [21,59,60]. Additionally, ecological changes resulting from ALAN can lead to increased disservices for humans. For example, changes in the abundance and behaviour of vector species (e.g. mosquitoes) owing to ALAN can impact the dynamics of vector-borne diseases such as malaria, as Coetzee *et al*. [61] show in this issue.

Understanding and addressing these multifaceted impacts of ALAN on ecosystems, humans and their interconnectedness is crucial for developing effective strategies to mitigate light pollution. Evans [32] notes that although mitigation options are numerous, few have been rigorously tested. Given that light pollution impacts strongly vary among species [17], this suggests that mitigation strategies may not be universally applicable. For example, turning off lights for part of the night—a potential mitigation strategy—might actually lead to a decrease in herbivore population growth compared to constant all-night illumination as shown in Heinen *et al*. [31]. Additionally, in a long-term study, Spoelstra *et al*. [29] show that while light, in general, shifted terrestrial invertebrate community compositions, the specific effect of different light spectra was not conclusive.

The impact of mitigation strategies may be different when considering entire ecosystems, thereby potentially enhancing the overall applicability of these mitigation approaches. For example, Hölker *et al*. [51] provide a strong call to action to move towards environmentally friendly electric lighting in areas near freshwater ecosystems, which are sensitive to light disturbance. Additionally, they recommend considering not only the ecosystems at the greatest risk, but interconnected landscapes and systems that may also be impacted by ALAN.

Overall, by recognizing the interactions and feedback loops involved in complex ecological systems, we can strive for a balance between the benefits of artificial lighting for humans and minimizing its negative consequences, fostering sustainable coexistence between humans and the natural environment.

## Data Availability

This article has no additional data.
